# Introduction to the *RSC Advances* themed collection on metal extraction and recycling

**DOI:** 10.1039/d3ra90107f

**Published:** 2023-11-16

**Authors:** Isabelle Billard, Alexandre Chagnes, Euan Doidge, Jason B. Love, Magdalena Regel-Rosocka

**Affiliations:** a Université Grenoble Alpes, LEPMI 1130 Rue de la Piscine Saint Martin d'Hères 38402 France Isabelle.Billard@grenoble-inp.fr; b University of Lorraine, GeoRessources 2 Rue du Doyen Marcel Roubault Vandoeuvre les Nancy 54500 France; c Imperial College London Chemistry Building, South Kensington Campus UK; d The University of Edinburgh, EaStCHEM School of Chemistry Joseph Black Building, David Brewster Road, The King's Buildings Edinburgh EH9 3FJ UK; e Poznan University of Technology Poznan 60-965 Poland

## Abstract

Professor Isabelle Billard, Professor Alexandre Chagnes, Dr Euan Doidge, Professor Jason Love and Professor Magdalena Regel-Rosocka, introduce this *RSC Advances* themed collection on metal extraction and recycling.
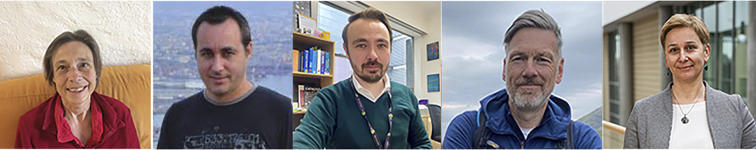

Extraction of metal ions is a long-standing and well-established area of science with tremendous success to date, for example in ore processing or nuclear waste management, and many other applications directly connected to our everyday life. In this sense one may think there is nothing more to prove or publish, but this themed collection, entitled “Metal extraction and recycling”, will certainly convince any reader that this remains a vibrant field of research, helping to address the major new challenges this scientific community is facing. First, the scarcity of metal resources is forcing ever-better efficiencies, in more and more complex ore matrixes that are also of lower grade. Second, the demand for less common metals calls for new extraction systems. Third, the impulse towards recycling of discarded technological objects (*e.g.* mobile phones, computer screens, batteries, photovoltaic panels and now, even connected socks!) has drastically modified the type of starting material that can be used, displaying uncommon mixtures of glass, ceramics, plastics and, of course, unusual, and ever changing, metal compositions. Finally, the European REACH regulation^1^ now demands more eco-friendly processes.

This themed collection is an excellent demonstration of successful efforts to solve complex extraction and recycling problems. We would first like to emphasize that metal extraction experiments do not rely only on classical batch experiments but take advantage of numerous modern spectroscopic and diffraction techniques, as well as advanced mathematical and computational tools to study system thermodynamics, extraction mechanisms and kinetics, often in real industrial samples. Used lithium batteries are a typical example of the highly complex systems at the heart of metal extraction studies in this themed collection, even without considering other costly parts of these wastes such as graphitic carbon (https://doi.org/10.1039/d2ra07926g). Industrial waste such as that from pickling baths, requires the recycling of metals such as zinc and iron to ensure pilot-scale circularity and to assuage environmental concerns (https://doi.org/10.1039/d2ra08195d). More fundamental studies are not to be outdone here, because a comprehensive knowledge of aqueous speciation is a prerequisite to efficient extraction, either using classical liquid–liquid processes or more original ones, such as magnetic crystallization or micellar adsorbents. As guest editors of the themed collection, we are proud of the diversity of subjects covered in this collection, and would like to thank all the authors for the quality of their work as well as the Royal Society of Chemistry staff for the quality of the presentation.

However, we have not yet covered all ground, and the issues that still need to be tackled head-on should be highlighted. Being able to extract metals and other materials is an important task but effectively recycling used metals into new devices should be an even more important goal; a recyclable phone is good, but a phone made from recycled materials is even better. One way to achieve this is to ensure that Life Cycle Analysis (LCA) and eco-design principles are embedded into future extraction systems from the outset. This certainly paves the way for exciting future works, making the best of a broad variety of fundamental and applied physico-chemical studies, and involving a variety of knowledge, competences, and skills that only exist within collaborative teams. Keep an eye out for future developments in the field!

## Supplementary Material
